# Supplementary material for: Impact of the COVID-19 pandemic on excess maternal deaths in Brazil: A two-year assessment
Source: PLoS One. 2024 Apr 2;19(4):e0298822. doi: 10.1371/journal.pone.0298822 (PMC10986939; doi:10.1371/journal.pone.0298822)
Supplement: S1 Table — (DOCX) [file pone.0298822.s001.docx]

**S1 Table.** Description of causes of death among victims of maternal deaths, by each block of chapter XV of the International Statistical Classification of Diseases and Related Health Problems (ICD) 10th Revision, March 2020 to May 2021, Brazil.

| ICD - Blocks | Description | n | (%) |
| --- | --- | --- | --- |
| O85-O99 | Complications predominantly related to the puerperium and other obstetric conditions, not elsewhere classified | 3,273 | 64.9% |
|  |  |  |  |
| O10-O16 | Oedema, proteinuria and hypertensive disorders in pregnancy, childbirth and the puerperium | 680 | 13.5 |
|  |  |  |  |
| O20-O29, O60-O63, O67-O71, O73-O75, O81-O84 | Other complications of labour and delivery | 415 | 8.2 |
|  |  |  |  |
| O72 | Postpartum haemorrhage | 228 | 4.5 |
|  |  |  |  |
| O00-O02, O05-O08 | Other pregnancies which ended in abortion | 220 | 4.4 |
|  |  |  |  |
| O44-O46 | Placenta praevia, Premature separation of placenta and Antepartum haemorrhage, not elsewhere classified | 128 | 2.5 |
|  |  |  |  |
| O30-O43, O47-O48 | Other reasons of maternal care related to the fetus and amniotic cavity and possible delivery problems | 58 | 1.2 |
|  |  |  |  |
| O03 | Spontaneous abortion | 33 | 0.7 |
|  |  |  |  |
| O04 | Medical abortion | 3 | 0.1 |
|  |  |  |  |
| O64-O66 | Obstructed labour | 2 | 0.1 |
|  |  |  |  |
| Total | Pregnancy, childbirth and the puerperium | 5,040 | 100% |
