# Supplementary material for: Impact of the COVID-19 pandemic on excess maternal deaths in Brazil: A two-year assessment
Source: PLoS One. 2024 Apr 2;19(4):e0298822. doi: 10.1371/journal.pone.0298822 (PMC10986939; doi:10.1371/journal.pone.0298822)
Supplement: Attachment — Submitted filename: Resposta_Pareceres_PONE-D-20231126 DL.docx [file pone.0298822.s003.docx]

ACADEMIC EDITOR:

Summary

Highlight which statistical method was used in the analyses and the R libraries.

RESPONSE: We appreciate your recommendation; information pertaining to the packages used during statistical modeling was added to the abstract, as follows: “*Analyses were performed in R version 4.1.2, RStudio, version 2023.03.1+446 and carried out with support from the “mgcv” and “plot_model” libraries.”* This change is also reflected in the Methods section (lines 231-233).

Introduction

The authors should discuss the contributions of this manuscript with the one previously published in 2022: Orellana J, Jacques N, Leventhal D.G.P., Marrero L, Morón-Duarte LS. Excess maternal mortality in Brazil: Regional inequalities and trajectories during the COVID-19 epidemic. PLoS One. 20 Oct 2022;17(10):e0275333).

RESPONSE: We appreciate the suggestion and agree that the Introduction can be improved by highlighting these analyses’ contribution in relation to the previous article on excess maternal mortality in Brazil. We added the following text to the Introduction section: “*A previous study examining data from March 2020 to May 2021 estimated a 70% excess in maternal mortality in Brazil, with wide variations by region and time period evaluated [2]. However, the study did not allow for an evaluation of the possible indirect effects of the COVID-19 epidemic on maternal mortality in traditional age groupings (10-19 years, 20-34 years, and 35-49 years). In addition, the study did not assess excess maternal deaths in the second and most critical year of the epidemic in different regions of Brazil, especially in the months following the late, uninterrupted distribution of vaccines against COVID-19 to pregnant and post-partum women beginning in July 2021.”*

Methodology

• Evaluate the data quarterly to dialogue with the manuscript (Orellana J, Jacques N, Leventhal D.G.P., Marrero L, Morón-Duarte LS. Excess maternal mortality in Brazil: Regional inequalities and trajectories during the COVID-19 epidemic. PLoS One Oct 20, 2022;17(10):e0275333).

RESPONSE: We thank you for your pertinent comment. Nevertheless, we have chosen to maintain the analysis by quadrimester because 1) the overall estimates from Years 1 and 2 will be the same both in analyses by trimester and by quadrimester, and, in particular, 2) the analysis by quadrimester improves the accuracy of our predictions (especially those related to maternal deaths in women aged 10-19 years, in regions or even in other subanalyses within regions with a smaller “n,” like the Central-West and South regions) and, finally, 3) the quadrimester analysis simplifies the presentation and discussion of the results of the two-year assessment. The rationale for this decision is provided in the Methods section, as follows: “*The chosen level of aggregation (by quadrimester) was agreed upon with the aim to improve the accuracy of our predictions, especially those pertaining to maternal deaths in women aged 10-19 years or even in subanalyses in regions with smaller samples, like the Central-West and South regions.”*

Present ICD10 codings in a table to facilitate reading.

RESPONSE: Thank you and we agree with your suggestion to include a table, substituting the previous description in the text. The table is entitled “**Table 1.** Maternal deaths according to codings from the International Statistical Classification of Diseases and Related Health Problems, 10^th^ Revision (ICD-10)”.

**Table 1.** Maternal deaths according to codings from the International Statistical Classification of Diseases and Related Health Problems, 10^th^ Revision (ICD-10)”.

| **ICD-10 Codings** | **Group of maternal deaths** |
| --- | --- |
| O00-O99 | Pregnancy, childbirth and the puerperium* |
| B20-B24 | Human immunodeficiency virus [HIV] disease** |
| D39.2 | malignant or invasive hydatidiform mole** |
| E23.0 | Hypopituitarism** |
| M83.0 | Puerperal osteomalácia*** |
| A34 | Obstetrical tetanus*** |
| F53 | Mental and behavioural disorders associated with the puerperium, not elsewhere classified*** |

*Except deaths outside of the 42-day postpartum period (codes O96 and O97)

**With a gestational report at the time of death or up to 42 days before death

***With death occurring up to 42 days after the termination of pregnancy or when there was no information on the time elapsed between the termination of gestation and death

• Was any methodology applied to correct deaths? If not, what is the reason?

LAURENTI, R.; MELLO JORGE, M.H.P. of M.; GOTLIEB, S. L. D. Maternal mortality in Brazilian capitals: some characteristics and estimates of an adjustment factor.Rev. Brazilian Epidemiology, São Paulo, v. 4, p. 449-460, Dec. 2004. Available at: http://www.scielo.br/pdf/rbepid/v7n4/08.pdf.

LUIZAGA, C.T.M. et al. Maternal deaths: revision of the correction factor for official data. Epidemiol. Serve. Health, Brasília, v. 1, pg. Mar 8 to 15th. 2010. Available at: http://scielo.iec.gov.br/pdf/ess/v19n1/v19n1a02.pdf. Accessed on: Mar 13, 2018

RESPONSE: We appreciate your important suggestion and for the purposes of clarification have included the reason for not using the method of correcting maternal deaths in the revised version of the Methods section. The altered text is as follows: “*We analyzed data from March 2020 to February 2022. We did not apply correction factors to the maternal mortality estimates, as the focus of the article is to present and discuss excess deaths as a percentage. These do not change, with or without a fixed correction on the observed or estimated counts.”*

• Present the formulas of the statistical models used.

RESPONSE: We thank you for your important suggestion. We have included our formula with a textual explanation of each variable used in the Methods section (lines 187-213).

Discussion

Highlight the similarities and differences between this article and the previous manuscript (Orellana J, Jacques N, Leventhal D.G.P., Marrero L, Morón-Duarte LS. Excess maternal mortality in Brazil: Regional inequalities and trajectories during the COVID-19 epidemic PLoS One. Oct 20, 2022;17(10):e0275333).

RESPONSE: We are grateful for and agree on your timely suggestion. For this reason, we have added to the Discussion section, highlighting the contributions of the current version of the article assessing excess maternal mortality in Brazil, as follows: *“Compared to a previous analysis on excess maternal mortality in Brazil [2], this study not only confirms fears that the percentage of excess maternal deaths in Year 2 of the COVID-19 epidemic would be even larger compared to Year 1, but also provides documentation of the unexpected negative impact in the South region of the country in Year 2 and of the slight difference in the percentage of excess maternal deaths in the North region between Years 1 and 2. It is also worth highlighting that we presented estimates by age groups that are more easily comparable with the maternal mortality literature, in addition to more accurate predictions in subanalyses with smaller sample sizes, since our minimum temporal aggregation is now four-month rather than three-month periods, as was our initial approach [2].”*

Conclusion

It is too short. Authors need to indicate the contributions of their results to public health assessment and planning. What are the factors that develop into the exposed reality? Moreover, it will make it challenging to achieve the goals of the Sustainable Millennium Goals.

RESPONSE: We thank you for and agree with this crucial suggestion. For this reason, we have added the following text: “*We cannot emphasize enough the importance of a timely and effective epidemic response, not only to prevent tragic, avoidable deaths during acute periods, but also to counter the residual effects of the COVID-19 pandemic and of new crises in a manner that is sensitive to low-and middle-income country contexts, such as Brazil’s clear distancing from its commitment through the Sustainable Development Goals (SDGs) to reduce maternal mortality to at most 30 cases per 100 thousand live births by 2030*.”

Reviewer #1:

This manuscript is essentially an updated version of an article published a year ago (Orellana J, Jacques N, Leventhal DGP, Marrero L, Morón-Duarte LS. Excess maternal mortality in Brazil: Regional inequalities and trajectories during the COVID-19 epidemic. PLoS One. 2022 Oct 20;17(10):e0275333). Its publication could be justifiable by the fact of adding to the analysis presented in the previous work the period between June 2021 and February 2022, still relevant for the COVID-19 pandemics in Brazil. However, it would be helpful if it kept the same trimestral time division and the same structure of the paper published, in order to “talk” more directly and easily with it.

RESPONSE: Thank you for your suggestions. Please see our responses to the academic editor’s suggestions dealing with the Introduction and Methods sections.

The option for presenting only one table with data for all regions and the country e much better than the option chosen in this version.

RESPONSE: Thank you for the suggestion. However, the results from our second article would be disproportionally represented in one table, as we are dealing with data from Years 1 and 2 as well as their overall distribution for Brazil and all its regions, unlike in our first article. For this reason, we have decided to present results in more than one table.

The organization of the results’ text by also sounds better in the previous article.

RESPONSE: We appreciate the comment, but we have chosen to maintain the current organization of the results, due to the larger volume of data being presented.

I would recommend that the authors make more explicit the connection of the work with the previous one, underlining why the updated version was done.

RESPONSE: Thank you for your suggestion. Please see our responses to the academic editor for the Introduction and Discussion sections.

Perhaps some background and methodological elements may be synthesized, referring that more information is in the other article.

RESPONSE: We appreciate the recommendation. We have opted to provide more clarity to certain parts of the Methods section as well as to synthesize part of the text in a table.

If both articles are similar, it better that their similarities and complementarities are clear.

RESPONSE: Thank you for your suggestion. Please see our reply to your penultimate suggestion.

The text could be reviewed to become more fluid; some style repetition makes it a little boring.

RESPONSE: We thank you for your constructive comment and believe that the revised version of the present article has made the text clearer and more fluid.

Small details such as using “from 2018-2019” should also be avoided.

RESPONSE: Thank you for the constructive commentary. We have made the appropriate changes.

The authors may chose between “from 2018 to 2019” or “in (the period) 2018-2019.

RESPONSE: Thank you for your helpful suggestion. We have changed “2018-2019” to “2018 to 2019.”
