# Supplementary material for: Impact of the COVID-19 pandemic on excess maternal deaths in Brazil: A two-year assessment
Source: PLoS One. 2024 Apr 2;19(4):e0298822. doi: 10.1371/journal.pone.0298822 (PMC10986939; doi:10.1371/journal.pone.0298822)
Supplement: Attachment — Submitted filename: Resposta_20240121 DL.docx [file pone.0298822.s004.docx]

ACADEMIC EDITOR:

The manuscript requires minor adjustments to the table titles and the model formula, specifically incorporating the random error, which is currently missing.

We thank the editor for their comments. Regarding the subject of the formula we provided, we have opted to clarify a few points about the quasipoisson generalized additive model (GAM) instead of including the random error, as follows in lines 155-161: “The mean parameter θ specifically is used to address overdispersion (when the variance of count data is larger than the mean) and does not have a specific associated distribution, acting as a scaling factor that adjusts the variance to reflect the observed overdispersion. Therefore, the modeled distribution is proportional to the mean estimated by the model, expressed mathematically as Var(Y∣X)= ø ⋅ µ, where µ is the expected mean. Furthermore, when the value of ø is larger than one, this indicates that the data display more variability than expected (assuming a *Poisson* distribution in which both the mean and the variance are equal).”

We have also changed the title of tables 2-4 to reflect the information contained therein (e.g., Table 2. Observed and expected maternal deaths, Brazil and Southeast region, 2020-2022).

Reviewer #1:

The questions raised previously were satisfactorily addressed. My only note at this point is that the titles of Table 2, 3 and 4 need to be appropriately defined.

We are grateful for the attention given to this matter. Accordingly, we have named Tables 2-4 based on the information they present, as mentioned above in the response to the editor.
